# Surface tension of Nanofluid-type fuels containing suspended nanomaterials

**DOI:** 10.1186/1556-276X-7-226

**Published:** 2012-04-18

**Authors:** Saad Tanvir, Li Qiao

**Affiliations:** 1 School of Aeronautics & Astronautics, Purdue University, West Lafayette, IN, 47907, USA

**Keywords:** Nanofluids, Surface tension, Van der Waals force, Electrostatic force

## Abstract

The surface tension of ethanol and n-decane based nanofluid fuels containing suspended aluminum (Al), aluminum oxide (Al_2_O_3_), and boron (B) nanoparticles as well as dispersible multi-wall carbon nanotubes (MWCNTs) were measured using the pendant drop method by solving the Young-Laplace equation. The effects of nanoparticle concentration, size and the presence of a dispersing agent (surfactant) on surface tension were determined. The results show that surface tension increases both with particle concentration (above a critical concentration) and particle size for all cases. This is because the Van der Waals force between particles at the liquid/gas interface increases surface free energy and thus increases surface tension. At low particle concentrations, however, addition of particles has little influence on surface tension because of the large distance between particles. An exception is when a surfactant was used or when (MWCNTs) was involved. For such cases, the surface tension decreases compared to the pure base fluid. The hypothesis is the polymer groups attached to (MWCNTs) and the surfactant layer between a particle and the surround fluid increases the electrostatic force between particles and thus reduce surface energy and surface tension.

## Background

Nanofluids are liquids with stable suspension of nanometer sized particles (1–100 nm). The nanoparticles used in nanofluids are typically made of metals, oxides, carbides, or carbon nanotubes. Studies from the past decade show that this innovative class of composite fluid exhibit much higher thermophysical properties such as thermal conductivity and diffusivity as compared to the base fluid, and thus can be used for more effective cooling or heating for various thermal and energy applications [1-3].

Recently, the combustion and propulsion community has increasing interest in developing high-performance nanofluid-type fuels. The idea is to suspend nanomaterials (such as nanoenergetic particles and nanocatalysts) in traditional liquid fuels to enhance performance. Previous studies have shown nanofluid fuels with the addition of energetic nanomaetierals such as aluminum and boron and nanocatalyst such cerium oxide have shown promising performance [4-11], e.g., higher energy release, shortened ignition delay, increased burning rate, increased ignition probability, and enhanced catalytic effect.

While several studies have explored the combustion behavior of nanofluid-type fuels, their physical properties such as viscosity and surface tension have rarely been studied. Especially, surface tension, which is defined as the force acting over the surface of the liquid per length of the surface perpendicular to the force [2], is an important parameter. For instance, surface tension has a significant impact on boiling process as bubble departure and interfacial equilibrium depends on it [2,12,13]. The wetting behavior of nanofluids is of particular interest to the microfluidics community, in which surface tension plays an important role. And a reduction in surface tension leads to an enhancement of wettability of the fluid [14-16]. For combustion as well as pharmaceutical and paint coating applications spray characteristics such as droplet size, distribution and spray angle largely depend on surface tension.

However, there exists contradiction in the literature on the effects nanoparticle addition has on the surface tension of nanofluids as compared to the base fluids. Moosav et al. [17] using the Du Nouy Tensiometer demonstrated that the surface tension of base fluid (ethylene glycol) increases by a little over 7% with the addition of 3.0 vol.% ZnO nanoparticles. The authors attribute this to the accumulation of nanoparticles at the surface of the base fluid [17]. Golubovic et al. [12] using the capillary tube method showed that for low Al_2_O_3_ and BiO_2_ nanoparticle concentrations, there is very little deviation of surface tension from that of the base fluid, water. In a similar study by Kim et al. [13] employing the sessile drop method, it was observed that the surface tension started to increase after the addition of 0.01 vol.% alumina nanoparticles. Murshed et al. [18] and Kumar et al. [19] both showed that surface tension of carbon nanotube based nanofluids was higher than that of the base fluid, water. An opposite trend, however, was observed when Murshed et al. [20] tested TiO_2_/water nanofluids using a surface tensiometer. They showed that the addition of TiO_2_ to water reduced the surface tension of the resulting nanofluid at room temperature from that of water. The authors believe this reduction in surface tension is attributed to the Brownian motion and the adsorption of nanoparticles at the interfaces. Additionally, a study by Vefaei et al. [21] with Bi_2_Te_3_/water nanofluids by means of a sessile drop method showed that the surface tension decreased with increasing particle concentration until it reached a minimum and then increased with increasing particle concentration. The authors believe accumulation of nanoparticles at the gas-liquid interface to be responsible for the surface tension behavior. Furthermore, for most studies involving nanofluids, a surfactant or dispersant is necessary to be added to the mixture to obtain stable nanofluids. This may also influence surface tension. Recent work of Chen et al. [16] shows that adding surfactant, PVP (polyvinylpyrrolidone), does not affect the surface tension of the nanofluid. The reduction in surface tension can be attribution to the addition of silver nanoparticles rather than the PVP surfactant. However, the results in Ref. [18] and Ref. [19] show that the addition of NaBDS (sodium dodecyl benzenesulfonate) surfactant reduces the surface tension of DI water and DI water based nanofluids with the addition of carbon nanotubes. It is also noted that for lower concentrations of surfactant, the surface tension of the nanofluid remains unchanged.

In summary, there are contradictory conclusions regarding the changes of surface tension as a result of addition of nanoparticles. It is not clear at the moment whether the surface tension will increase or decrease and what mechanisms are responsible for such behavior. This is the motivation of the present paper. The objective is to determine the effect of particle addition (including particle material, size and concentration) on the surface tension of ethanol and n-decane based nanofluid fuels. The nanomaterials considered in this study include Al, B, Al_2_O_3_, and MWCNTs. The former two are energetic metals with high energy density; the latter two are potential catalysts.

## Methods

### Sample preparation

Preparation of the nanofluid fuels is based on the procedure developed by Gan et al [6]. An ultrasonic disruptor, which generates a series of 4-second-long pulses 4 seconds apart, was used to disperse particles evenly in the base fuel and to minimize agglomeration. Sonication was performed in an ice bath to maintain a constant temperature for the mixture. In addition to sonication, chemical stabilization by use of a surfactant was also adopted in some cases to reduce particle aggregation and to enhance stability of the suspensions.

**Figure 1:**
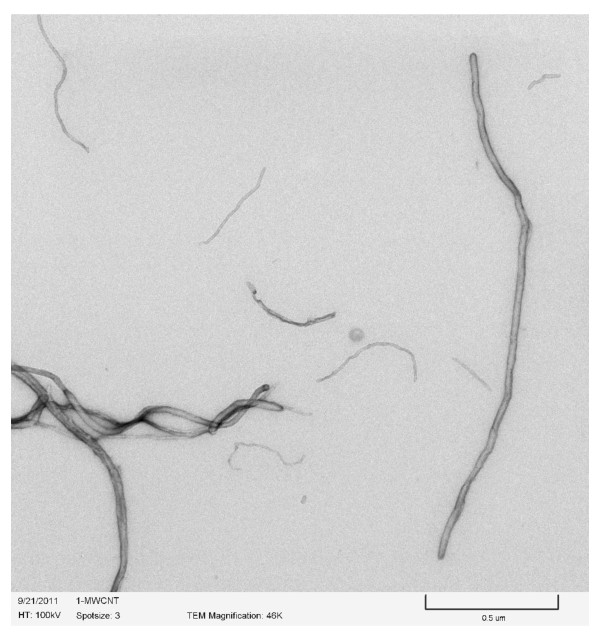
TEM image of multi-wall carbon nanotubes (MWCNTs) (D = 8–15 nm, L = 10–50μm).

**Figure 2:**
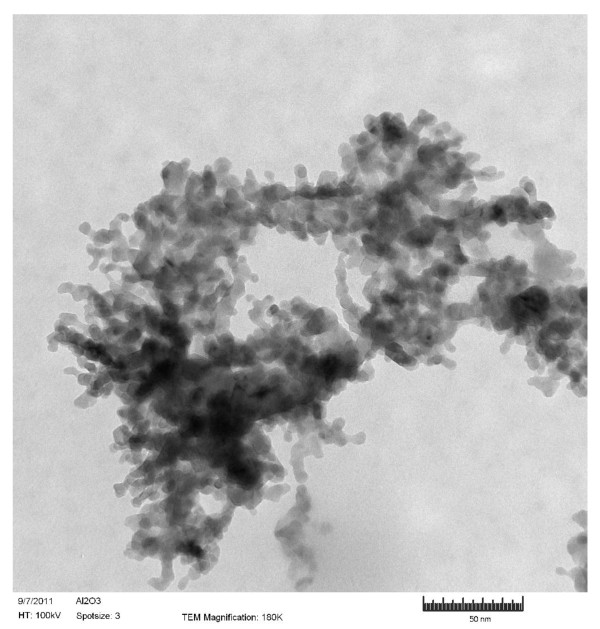
**TEM image of Al**_**2**_**O**_**3**_**nanoparticles (D = 25nm).**

**Figure 3:**
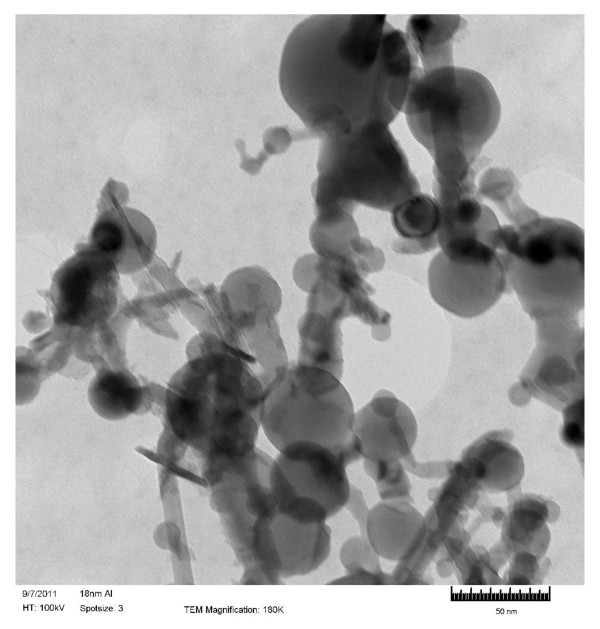
TEM image of Al nanoparticles (D = 18 nm).

**Figure 4:**
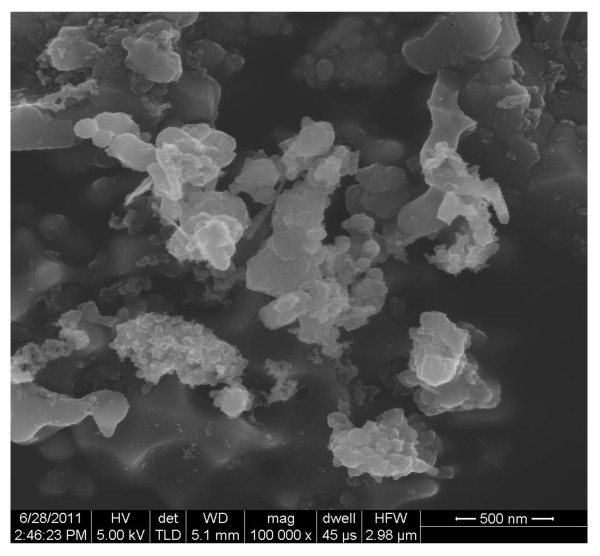
SEM image of B nanoparticles (D = 80 nm).

Ethanol and n-decane were the base fuel considered in the present study. Deionized (DI) water was also considered; the purpose was to compare the results to the literature, for which most nanofluids studies use water as a base fluid. Al_2_O_3_, Al, and B nanoparticles and MWCNTs, all purchased from Nanostructured and Amorphous Materials Inc, were added to the base fuel at concentrations of 0.1, 0.5, 1, 2, 3, 4, 5, 7 and 10 % by weight. Figures 1, 2, 3& show the TEM (Transmission Electron Microscopy) images of MWCNTs, Al_2_O_3_ and Al respectively. Figure 4 shows the SEM (Scanning Electron Microscope) image of B nanoparticles. The dispersible MWCNTs has an average diameter of 8–15 nm and a length of 10–50 μm. They comprised of the following components: 50–60 wt.% MWCNT, 33–43 wt.% polymers, 3.5 wt.% metals (Fe, Ni, La, Al, Si), 0.5 wt.% non-metals (Cl, S) and 3.0 wt.% amorphous carbon. The Al_2_O_3_ nanoparticles have an average size of 25 nm. The B and Al nanoparticles have an averaged diameter of 80nm and 18nm, respectively.

For water based nanofluids, only Al_2_O_3_ and MWCNTs were considered; and the purpose was to compare to existing data in the literature. In general, ethanol-based nanofluids have much better suspension quality than n-decane-based nanofluids, due to ethanol’s increased wettability. MWCNTs were suspended well in water and ethanol without the need of a surfactant; however they did not disperse well in n-decane even with the aid of surfactant. Sorbitan Oleate, a typical surfactant used for metal or metal oxides/oil suspensions, was used to stabilize n-decane based nanofluids. The surface tension measurements were performed immediately after sonication; therefore it was assumed that the nanofluids are stable with minimum agglomeration at the time of testing.

### Surface tension measurements

**Figure 5:**
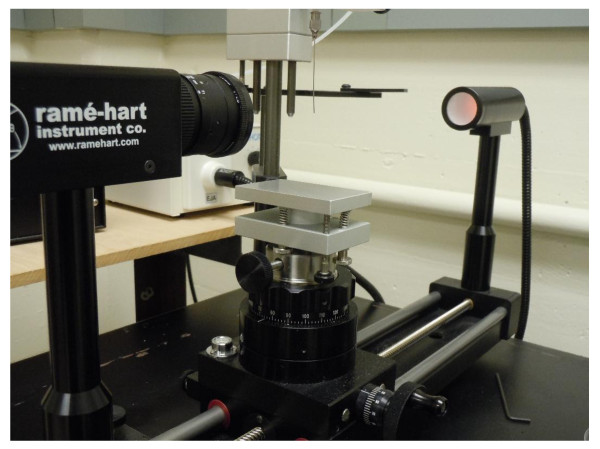
A picture of the Rame-Hart goiniometer setup.

A Rame-Hart Model 500 Standard goniometer (Figure 5) was used for real-time surface tension measurements. The pendant drop method was adapted to determine surface tension of the suspended droplet. The method utilizes the Young-Laplace equation to determine the surface tension of the droplet based on the shape of the droplet [22,23]. The droplet volume was controlled by a Rame-Hart automated dispensing system and varied between 6 and 12 μL. A 22 gage flat base needle was used to suspend the nanofluid droplet. Once a drop was suspended, the image was recorded by a camera and the image was then analyzed using the DROPimage advance software which calculated the surface tension using the Young-Laplace equation. Since the Young-Laplace equation calculates surface tension on bases of the difference in densities of the two phases (liquid-vapor), the densities of all the nanofluids were manually entered into the phase editor of the DROPimage advance software. The goniometer determines the contour and shape of the droplet with an accuracy of 1°.

The measurements were performed immediately after the droplet was suspended and stability had been established. This was to eliminate the effect of droplet vaporization during data acquisition. The measurement proved to be quantitatively accurate and highly repeatable. Four-five tests were performed for each nanofluid sample, and an average value was obtained from these tests. The measurements were repeatable within 1% of the mean for n-decane and DI water based nanofluids and 2% for ethanol based nanofluids. The needle and the dispensing system were regularly rinsed with DI water to prevent residue from prior experiments to impact measurements.

## Results and discussions

### Effect of particle concentration

**Figure 6:**
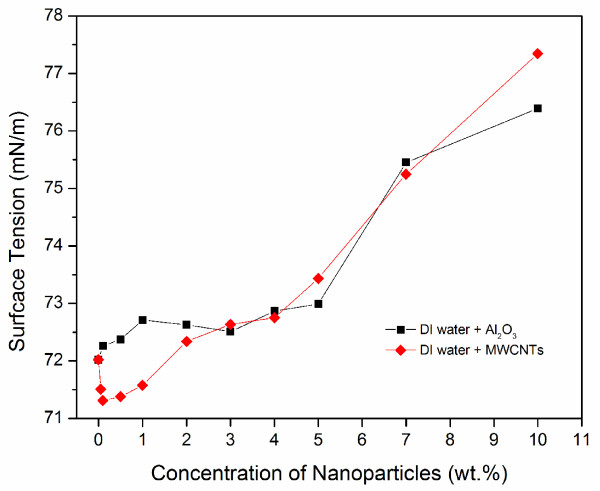
Surface tension variation with nanoparticle concentration for DI water based nanofluids.

We will first discuss the effect of particle concentration on surface tension. Figure 6 shows the variation of surface tension as a function of nanoparticle concentration (up to 10% by weight) of DI water containing Al_2_O_3_ and MWCNTs. For the Al_2_O_3_ nanofluid, the surface tension has little change (only very slightly increase) till 4 wt.%, which is consistent with the conclusion made by Kim et al [13]. After that, the surface tension increases with increasing concentration. Note that the surface tension of DI water at room temperature is 72.03 mN/m, which is comparable to the value reported in [20]. For the case of MWCNTs, it was found the surface tension initially decreases with particle concentration and then increases. At 10 wt.% MWCNT addition, the surface tension is about 7% higher than that of DI water.

**Figure 7:**
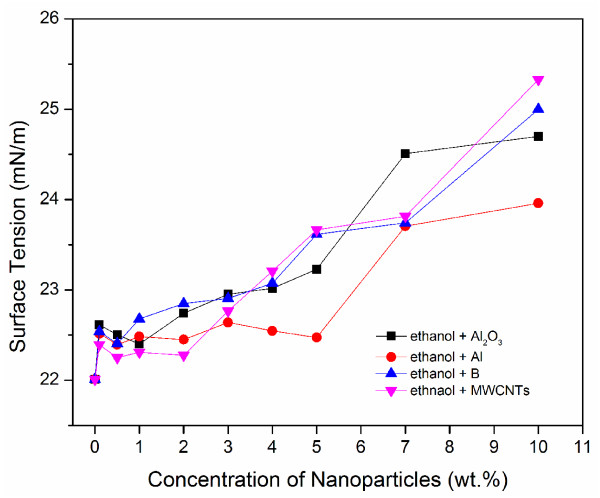
Surface tension variation with nanoparticle concentration for ethanol based nanofluids.

**Figure 8:**
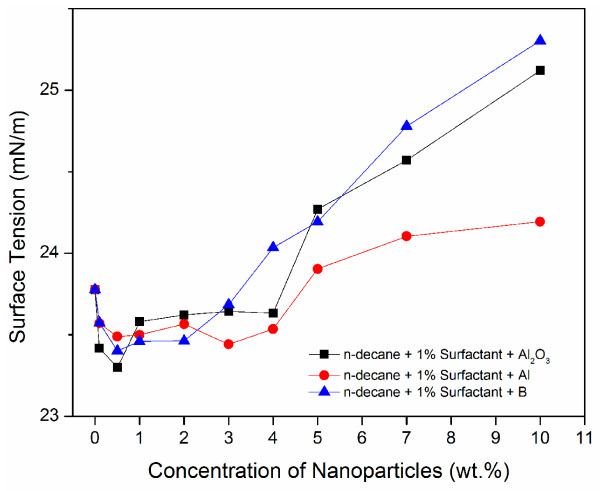
Surface tension variation with nanoparticle concentration for n-decane based nanofluids.

Figure 7 shows the surface tension of ethanol based nanofluids (including Al, Al_2_O_3_, B and MWCNTs). Note we did not add a surfactant to the mixtures because the suspension quality was quite good even without a surfactant. For all, the surface tension does not deviate much from that of pure ethanol for low particle concentrations up to 3 wt.% (a slight increase was observed); after which the surface tension increases with increasing particle concentration. For the n-decane based nanofluids, as shown in Figure 8, an initial decrease in surface tension was observed for nanoparticles up to 0.5% and remains almost constant up to 2–3 wt.%. After that, the surface tension increases with increasing particle concentration. Note for these nanofluids, 1 vol.% surfactant was added to promote chemical stability.

The experimental results clearly show that at high particle concentrations the surface tension will increases with particle concentration for all cases. At low particle concentrations, however, the trends are different for various base fluids, particles, with or without a surfactant. In the following, we will explain the observed trends based on how addition of nanoparticles could alter the surface energy at the liquid-gas interface, which will result in variation of surface tension. The nanoparticles tend to accumulate at the gas-liquid interface, indicating that the particle concentration at/near the liquid/gas surface will be higher than that inside the droplet. At the liquid/gas interface, the repulsive (electrostatic) and attractive forces (van der Waals) between particle, as well as a surfactant layer between a particle and the surrounding fluid molecules if a surfactant is being used, can potentially change the surface free energy [21].

For DI water containing Al_2_O_3_, the surface tension remains almost unchanged at low particle concentrations. This is likely because for such dilute suspensions, the distance between particles are much larger than the particle size, thus the forces and the interactions between particles at/near the liquid/gas interface has little impact on the surface energy. However, when the particle concentration increases, particles are getting closer to each other, thus the van der Waals force increases. This will increase the free energy at surface and results in higher surface tension. However, for DI water with the addition of small amount MWCNTs, as shown in Figure 6, a decrease in surface tension was observed. As mentioned earlier, dispersible MWCNTs used in this study are long with a length of 10–50 μm, although the diameter is small about 8–15 nm. Also they contain 33–43 wt.% polymers alongside the carbon nanotubes that aid in achieving stable nanofluids. It is possible that the electrostatic repulsion between the MWCNTs because of the polymer groups, which allows for good dispersion, reduces surface energy at the liquid-gas interface, and thus causes a reduction in surface tension. When the concentration of MWCNTs becomes high enough, the Van der Waals forces may dominate over the electrostatic repulsion force, and thus increase surface energy and surface tension.

Such explanation also applies to ethanol and n-decane based nanofluids, as show in Figures 7 and 8. As particle concentration increases, the mean spacing between the particles, especially at the liquid/gas interface, decreases. As a result, the attractive forces between the particles at the liquid-gas interface increases. As particle concentration increases, particle agglomeration increases resulting in a rise in surface tension. As a result, surface tension increases. This trend is clearly visible for all nanofluids considered in this study (Figures 6, 7, 8). However, at low particle concentrations, the phenomenon is more complicated. As mentioned earlier, there are discrepancies in the literature regarding whether the surface tension increases or decreases comparing to the base fluid. For the cases involving MWCNTs, as we have explained, the electrostatic repulsion because of the polymer groups, which allows for good dispersion, can reduce surface energy at the liquid-gas interface and thus reduce surface tension. When a surfactant is used, the surface tension tends to decrease at small particle concentration, while the surface tension does not change much when a surfactant is not used. We will discuss the effect of surfactant on surface tension in the next subsection.

### Effect of adding a surfactant

In the synthesis of n-decane nanofluids, a surfactant (Sorbitan Oleate) was added to the mixture to promote chemical stability of the suspension. The mechanism is called steric stabilization. The long chain surfactant molecules attach to the solid particle and form a layer between the particle and the surrounding fluid molecules. Such layers increase the potential between particles and impart a repulsive force between them. This in turn can reduce surface energy and thus surface tension.

**Figure 9:**
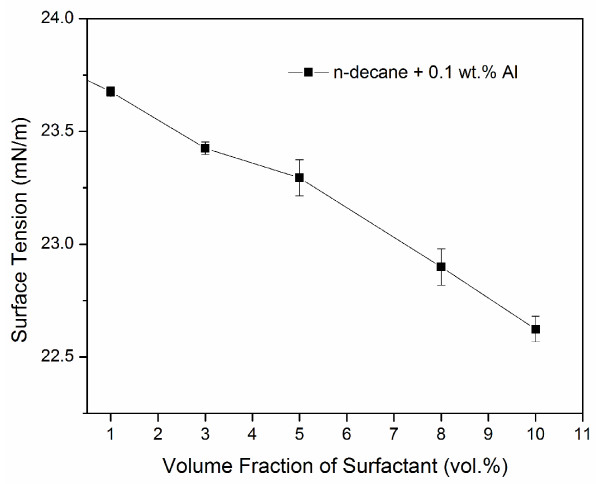
Surface tension variation with surfactant concentration for n-decane with 0.1 wt.% Al.

To test this hypothesis, we measured the surface tension of n-decane/surfactant/nano-Al mixtures. The Al nanoparticle concentration was kept the same at 0.1 wt.% but the surfactant concentration was varied between 1 and 10 vol.%. The results clearly show that with increasing volume fraction of the surfactant, the resulting surface tension of the nanofluid decreases (Figure 9). This is consistent with the literature that addition of surfactants to nanofluids tends to reduce the surface tension [18,19]. In particular, Vafaei et al. [21,24], who studied surface tension and contact angle variations of bismuth telluride nanofluids, attribute the changes in surface tension to the electrostatic interaction induced by the presence of the thioglycolic groups attached to the nanoparticles. The results, however, contradict with the findings in [16]. We believe the contradiction may arise from the use of different nanofluid/surfactant combinations in that the behavior of each type of nanofluid, upon addition of surfactant, will be different. In the present study the effect of droplet evaporation is negligible because the time interval between nanofluid preparations and testing is very small and because the duration of the test is less than 10 seconds. We believe the reduction in surface tension is attributed to the adsorption of ionic surfactant on the nanoparticle surface imparting an electrostatic repulsive force between the particles in the nanofluid. This electrostatic repulsion between nanoparticles surrounded by the surfactant at the liquid-gas interface results in the reduction of the surface tension of the nanofluid. This is consistent with the conclusions of Ref. [18] and Ref. [19], both of which show that for low surfactant concentrations, the surface tension remains unchanged and starts to decrease beyond a certain concentration level. The volume of surfactant added after which the surface tensions starts to decrease may indeed be labeled as excessive. The excessive surfactant may result in a further reduction in surface tension since its presence at the surface of the droplet is expected to increase.

### Effect of particle size

Another observation made from Figures 2, 3, 4 is that larger particles exhibit higher surface tensions at high concentrations of 5 wt.% and up (with the exception of 7wt.% aluminum oxide in ethanol). For both water and ethanol based nanofluids, the nanofluids containing MWCNTs (D = 8–15 nm, L = 10–50 μm) show the highest surface tension, followed by those of boron (80 nm), aluminum oxide (20–30nm) and then aluminum (18 nm). For n-decane based nanofluids, a similar trend was observed with boron nanofluids over 5 wt.% concentration have the highest surface tension followed by aluminum oxide and aluminum respectively. Such trend is likely due to the strengthened van der Waals force as particle size increases.

## Conclusions

The surface tension of DI water, ethanol and n-decane based nanofluids with addition of MWCNTs, B, Al, and Al_2_O_3_ nanoparticles were studied. The results show that at high particle concentrations, surface tension of the nanofluids increases with increasing particle concentration, as compared to that of the base fluids. This is likely due to the increasing Van der Waals force between the accumulated particles at the at the liquid-gas interface, which increases the surface free energy and cause the surface tension to increase. However, at low particle concentrations (below 3–4 wt.%), additional of particles generally has little influence on the surface tension because the distance between the particles is large enough even at the liquid/gas interface. An exception is for the nanofluids containing MWCNTs or when a surfactant is added to the nanofluids. In such cases, the surface tension decreases at low particle concentrations, compared to the pure base fluid. This is because of the electrostatic repulsive force between particles, which is present due to the existence of a surfactant layer or the polymer groups attached to MWCNTs, reduces the surface free energy and thus causes a reduction in surface tension. Lastly, the results show that surface tension decreases with increasing surfactant concentration, and increases with increasing particle size. A follow-up study is planned in the future to understand the impact of surfactant at low concentrations.

## Abbreviations

DI, deionized; L, liter; MWCNT, multi-walled carbon nanotubes; NaBDS, sodium dodecyl benzenesulfonate; PVP, polyvinylpyrrolidone; SEM, scanning electron microscopy; TEM, transmission electron microscopy; vol.%, volume percentage; wt.%, weight percentage.

## Competing interests

Both authors declare that they have no competing interests.

## Authors’ contributions

ST conducted experiments and analysis. LQ supervised the research. Both authors read and approved the final manuscript.

## Authors’ information

ST is a graduate research assistant at the School of Aeronautics and Astronautics, Purdue University, West Lafayette, IN, USA. LQ is an assistant professor at the School of Aeronautics and Astronautics, Purdue University, West Lafayette, IN, USA.
